# Didymin improves UV irradiation resistance in *C. elegans*

**DOI:** 10.7717/peerj.6218

**Published:** 2019-01-09

**Authors:** Lin Zhou, Lu Wang, Jialing Zhang, Jiahe Li, Shuju Bai, Junfeng Ma, Xueqi Fu

**Affiliations:** National Engineering Laboratory for AIDS Vaccine, School of Life Sciences, Jilin University, Changchun, Jilin Province, China

**Keywords:** Didymin, *Caenorhabditis elegans*, Lifespan, UV irradiation

## Abstract

Didymin, a type of flavono-o-glycoside compound naturally present in citrus fruits, has been reported to be an effective anticancer agent. However, its effects on stress resistance are unclear. In this study, we treated *Caenorhabditis elegans* with didymin at several concentrations. We found that didymin reduced the effects of UV stressor on nematodes by decreasing reactive oxygen species levels and increasing superoxide dismutase (SOD) activity. Furthermore, we found that specific didymin-treated mutant nematodes *daf-16*(mu86) & *daf-2*(e1370), *daf-16*(mu86), *akt-1*(ok525), *akt-2*(ok393), and *age-1*(hx546) were susceptible to UV irradiation, whereas *daf-2*(e1371) was resistant to UV irradiation. In addition, we found that didymin not only promoted DAF-16 to transfer from cytoplasm to nucleus, but also increased both protein and mRNA expression levels of SOD-3 and HSP-16.2 after UV irradiation. Our results show that didymin affects UV irradiation resistance and it may act on *daf-2* to regulate downstream genes through the insulin/IGF-1-like signaling pathway.

## Introduction

As the natural environment deteriorates and the pace of life accelerates, people receive more and more types of stimuli, such as smog, high temperature, radiation, noise, and so on. These stimuli destroy the balance between the body’s oxidation and antioxidation systems, which in turn causes the body to produce and accumulate large amounts of reactive oxygen species (ROS), eventually leading to oxidation reactions (Schieber & Chandel, 2014). Stress response could induce DNA damage, cell apoptosis and body’s immune response to affect daily behavior and health of the organism, involving many important cell signal transduction processes, such as insulin signaling pathway (Sadagurski & White, 2013). In recent years, the prevention and treatment of biological anti-stress has received increasing attention. Its mechanism research has become the focus of disease treatment and drug development.

The classic definition of oxidation states that oxygen-containing elements or compounds are combined for subsequent oxidation reactions. The term oxide was coined by French chemists Guyton DE Morveau and Antoine Lavoisier, and it is based on oxygen and acid. The oxidation and reduction processes occur together, and therefore they are collectively referred to as redox reactions (Sies, 2015). Antioxidants, including natural antioxidants and synthetic antioxidants, are substances that prevent other substances from being oxidized (Moreira, Villas Boas & Ferreira, 2014); in other words, they can inhibit or delay the oxidation process. The natural antioxidants are structurally diverse and abundant in the environment, and they have been extensively studied in the fields of drug discovery and development. Didymin is a dietary flavonoid (Morelli et al., 2014) found in citrus fruits such as lemons, oranges, and bergamot (Lin et al., 2016). It could lead non-small cell lung cancer cell to die in the form of p53 (Hung et al., 2010). In addition, it inhibited N-myc expression, stimulated the Raf kinase inhibiting protein level during neuroblastoma proliferation, and induced cell apoptosis, which is an effective new method to treat neuroblastoma (Singhal et al., 2012). Although didymin’s anti-cancer potential is well known, its roles in stress resistance have not been thoroughly investigated.

Nematodes are an excellent model organism (Ewald, 2018). Although seemingly simple, nematodes have a high degree of differentiation. They have muscles, subcutaneous tissues, nervous systems, intestines, gonads, glands, and excretory systems. Due to their small size, short life cycle, and high fertility, nematodes can be used in the screening for new drugs. In addition, nematodes are transparent; thus, they can be easily tracked and located in the body using fluorescent markers. Therefore, nematodes can be used to evaluate the efficacy, absorption, distribution, metabolism, excretion, and toxicity of drugs at the initial stages of drug discovery (O’Reilly et al., 2014). In this study, we investigated the effects of didymin on multiple stressors in *Caenorhabditis elegans*. It could not only be a benefit for improving screening of anti-UV irradiation drugs, but also to promote didymin research. Nematodes’ homologues have been identified for 60–80% of human genes (Kaletta & Hengartner, 2006); therefore, the results obtained by using the nematode model could provide a theoretical basis for human diseases treatment.

## Materials and Methods

### Chemicals and reagents

Didymin (C_28_H_34_O_14_; >98% pure, HPLC-grade) and dimethyl sulfoxide (DMSO) were taken from Dalian Meilun Biological Technology Co., Ltd. (Dalian, China). Juglone, levamisole and dichlorofluorescin diacetate (DCFH-DA) were bought from TransGen Biotech Co., Ltd. (Beijing, China). The superoxide dismutase (SOD) Activity Detection Kit was obtained from Beijing Solarbio Science and Technology Co., Ltd. (Beijing, China). The Easypure RNA Kit, TransScript One-Step gDNA Removal and cDNA Synthesis SuperMix, and TransStart Green qPCR SuperMix were purchased from TransGen Biotech Co., Ltd. (Beijing, China). All other chemicals were of analytical or reagent grade.

### *C. elegans* strain and cultivation

The strains used in this study were as follows: Bristol N2 (wild-type), CF1588 (*daf-16*(mu86) I; *daf-2*(e1370) III; muIs84), CF1038 (*daf-16*(mu86) I), RB759 (*akt-1*(ok525) V), VC204 (*akt-2*(ok393) X), TJ1052 (*age-1*(hx546) II), DR1568 (*daf-2* (e1371) III), TJ356 (zIs356 [*daf-16*p::*daf-16*a/b::GFP + rol-6]), CF1553 (muIs84 [(pAD76) sod-3p::GFP + rol-6]), and CL2070 (dvIs70 [hsp-16.2p::GFP + rol-6(su1006)]). All strains and *Escherichia coli* OP50 were obtained from the Caenorhabditis Genetics Center (CGC).

The worms were cultured on nematode growth medium (NGM, 1.7% agar, 2.5 μg/mL peptone, 50 mM NaCl, 25 mM potassium phosphate, pH 6.0, one mM MgSO_4_, one mM CaCl_2_, and five μg/mL cholesterol) plates containing *E. coli* OP50 at 20 °C, unless otherwise specified. Didymin was dissolved in DMSO to a stock concentration of 10 mM. The treatment plates contained different final concentrations of didymin (0.05, 0.1, 0.2, 0.5, and one mM in 0.2% DMSO) or control solvent (0.2% DMSO).

### Lifespan assay

Lifespan assays were performed as previously described (Zhou et al., 2018b). Lifespan assays were carried out by growing wild-type nematodes to the L4 stage on NGM plates containing *E. coli* OP50 in an incubator set at 20 °C. The nematodes were incubated in the presence of didymin (0.05, 0.1, 0.2, 0.5, and one mM) dissolved in 0.2% DMSO for 48 h, whereas the control nematodes were incubated with 0.2% DMSO. The nematodes were then transferred to fresh plates every day. The viable and non-viable nematodes were recorded daily until all animals died. Non-viable nematodes failed to move after gently prodding with a platinum wire. The day of birth was taken as the first day of their lives. Die vulva blowout, bagging or climbing out of the plate were noted. This experiment was performed three times, with at least 100 nematodes per group.

### Stress resistance assay

The feeding program for the stress resistance assay was the same as that for the lifespan assay, and the conditions were identical to those in a previous study (Zhou et al., 2017). Wild-type nematodes at the L4 stage were incubated with 0.1 mM didymin for 48 and transferred to new plates without didymin, immediately followed by three kinds of stressors. This experiment was performed three times, with at least 100 nematodes per group.

To assess UV irradiation stress resistance, nematodes were exposed to 254 nm of UV irradiation at a dose of 1,000 J/m^2^ and immediately transferred to an incubator set at 20 °C. The viable and non-viable nematodes were scored every 12 h until all animals died.

To assess thermal shock stress resistance, the nematodes were transferred from an incubator set at 20 °C to one set at 35 °C incubator. The viable nematodes were scored every hour until all animals died.

To assess juglone stress resistance, the nematodes were placed in 96-well plates which contained 200 μL S medium (0.01 mM cholesterol, 100 mM NaCl, and 50 mM potassium phosphate, pH 6.0) supplemented with 200 mM juglone in each well. The viable nematodes were observed at 20 °C every hour until all animals died.

### Determination of ROS level

To measure the intracellular ROS levels, nematodes were cultured in the presence or absence of 0.1 mM didymin for 48 h and transferred to new plates without didymin, immediately followed by exposure to 1,000 J/m^2^ UV irradiation. Wild-type *C. elegans* were then immediately collected and steeped in 200 μM DCFH-DA for 30 min at 20 °C. Five hundred nematodes were washed three times with M9 buffer (one mM MgSO_4_, 22 mM KH_2_PO_4_, 42 mM Na_2_HPO_4_, and 85 mM NaCl) and then transferred into a black 96-well plate. A fluorescent microplate reader (Infinite F200 PRO, Tecan, Switzerland) were used to measure ROS-related fluorescence at 485 nm excitation wavelength and 525 nm emission wavelength (Zhang et al., 2018). Relative fluorescence unit was used to reflect relative ROS levels by comparing between control and didymin-treated groups. This experiment was repeated for three times.

### Measurement of superoxide dismutase activity

To measure SOD activity, wild-type nematodes at the L4 stage were cultured on NGM plates with or without 0.1 mM didymin for 48 h and transferred to new plates without didymin, immediately followed by 1,000 J/m^2^ UV irradiation. The nematodes were collected in M9 buffer and washed three times. Then, they were resuspended in M9 buffer and homogenized in ice (Lee, Xing & Kim, 2017). SOD activity was measured according to a SOD activity detection kit by using Multimode Plate Reader (Infinite F200 PRO, Tecan, Switzerland). This experiment was conducted three times, with at least 500 nematodes per group.

### Mutants UV irradiation resistance assay

The experimental method in this section is consistent with the description in “stress resistance assay.” Mutants used in this section were *daf-16*(mu86) & *daf-2*(e1370), *daf-16*(mu86), *akt-1*(ok525), *akt-2*(ok393), *age-1*(hx546), and *daf-2*(e1371).

### DAF-16 nuclear localization and SOD-3 and HSP-16.2 protein expression

The age-synchronized nematodes TJ356, CF1553, and CL2070 expressed DAF-16::GFP, SOD-3::GFP, and HSP-16.2::GFP fusion protein, respectively. Considering the main localization of DAF-16::GFP in each worm, the distribution of DAF-16 was categorized as cytosolic, intermediate, or nuclear. The nematodes were treated with 0.1 mM didymin under normal conditions at 20 °C for 48 h and transferred to new plates without didymin, immediately followed by 1,000 J/m^2^ UV irradiation. A total of five mM levamisole was used to anaesthetize worms for 3 min, and then animals were put on a microscope slide which was coated with 2% agar (Rea et al., 2005). GFP was monitored with a fluorescent microscope (DMI 3000 B; Leica, Wetzlar, Germany) which was equipped with a GFP filter at 365 nm excitation wavelength and 420 nm emission wavelength was used to observe GFP (Asthana et al., 2015; Zhang, Lu & Zhou, 2015). Image-Pro Plus Software (Media Cybernetics, Bethesda, MD, USA) was used to quantify fluorescent levels. This experiment was performed three times, with at least 10 nematodes per group.

### Quantitative real-time polymerase chain reaction

Total RNA was obtained from nematodes synchronized at L4 stage (*n* = ∼500 per group) treated with or without 0.1 mM didymin for 48 h and transferred to new plates without didymin, immediately followed by 1,000 J/m^2^ UV irradiation using the Easypure RNA Kit. TransScript One-Step gDNA Removal and cDNA Synthesis SuperMix was used to synthesize cDNA. TransStart Green qPCR SuperMix (SYBR green) was used as the label which could calculate the relative fold change in quantitative real-time polymerase chain reaction (qPCR) according to comparative 2^−ΔΔCT^ method and *actin* mRNA was served as the housekeeping gene. The qPCR primers used in this study were as follows: *act-1* (5′-GTCATGGTCGGTATGGGACA-3′ and 5′-TTCGTAGATTGGGACGGTGT-3′), *daf-16* (5′-TTTCCGTCCCCGAACTCAA-3′ and 5′-ATTCGCCAACCCATGATGG-3′), *sod-3* (5-TTCGAAAGGGAATCTAAAAGAAG-3′ and 5′-GCCAAGTTGGTCCAGAAGATAG-3′), and *hsp-16.2* (5′-CTGCAGAATCTCTCCATCTGAGTC-3′ and 5′-AGATTCGAAGCAACTGCACC-3′). This experiment was performed in three times (Pfaffl, 2001).

### Statistical analysis

For lifespan and stress resistance assays, Kaplan-Meier method using Prism 5 Software (GraphPad Software, La Jolla, CA, USA) was used to analyze data. Mantel-Cox (log-rank) test was used to analyze statistical significance. One-way analysis of variance was used to analyze all other data. Results were presented as the mean ± standard error of the mean. *p*-Values < 0.05 were taken as statistically significant (***p < 0.001; 0.001 ≤ **p < 0.01; 0.01 ≤ *p < 0.05).

## Results

### Didymin extends the lifespan of nematodes under normal culture conditions

**Figure 1:**
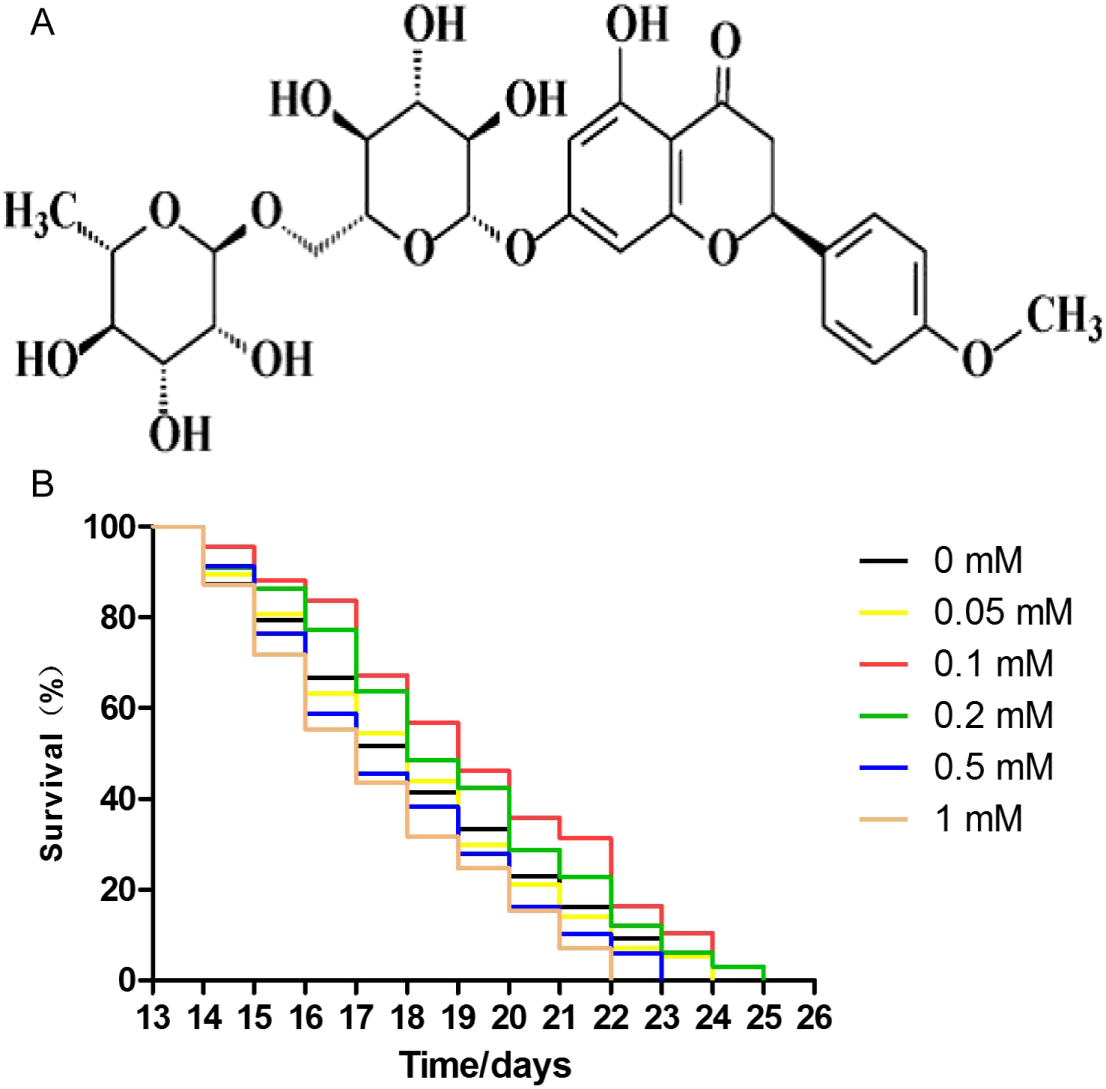
Didymin extended the lifespan of *C. elegans* wild-type nematodes under normal culture conditions. The chemical structure of didymin (A) is shown. (B) The adult worms were treated with didymin (0.05, 0.1, 0.2, 0.5, and one mM) or 0.2% DMSO (vehicle control). The greatest effect of didymin on the lifespan of nematodes occurred at 0.1 mM (*n* > 100, *p* < 0.001).

The effect of didymin on the lifespan of wild-type worms was measured. The concentrations of didymin used in this experiment were 0.05, 0.1, 0.2, 0.5, and one mM. Comparing with control worms treated with DMSO, lifespan of nematodes was increased by 0.05, 0.1, and 0.2 mM didymin in a dose-dependent manner. At concentrations of 0.5 and 1 mM, however, didymin decreased the lifespan of nematodes. Because didymin at a concentration of 0.1 mM prolonged the lifespan of nematodes most obviously, this dose was used for the remaining experiments which tested its protective effects on nematodes exposed to different stressors (Fig. 1).

### Didymin improves stress resistance of wild-type nematodes exposed to several stressors

**Figure 2:**
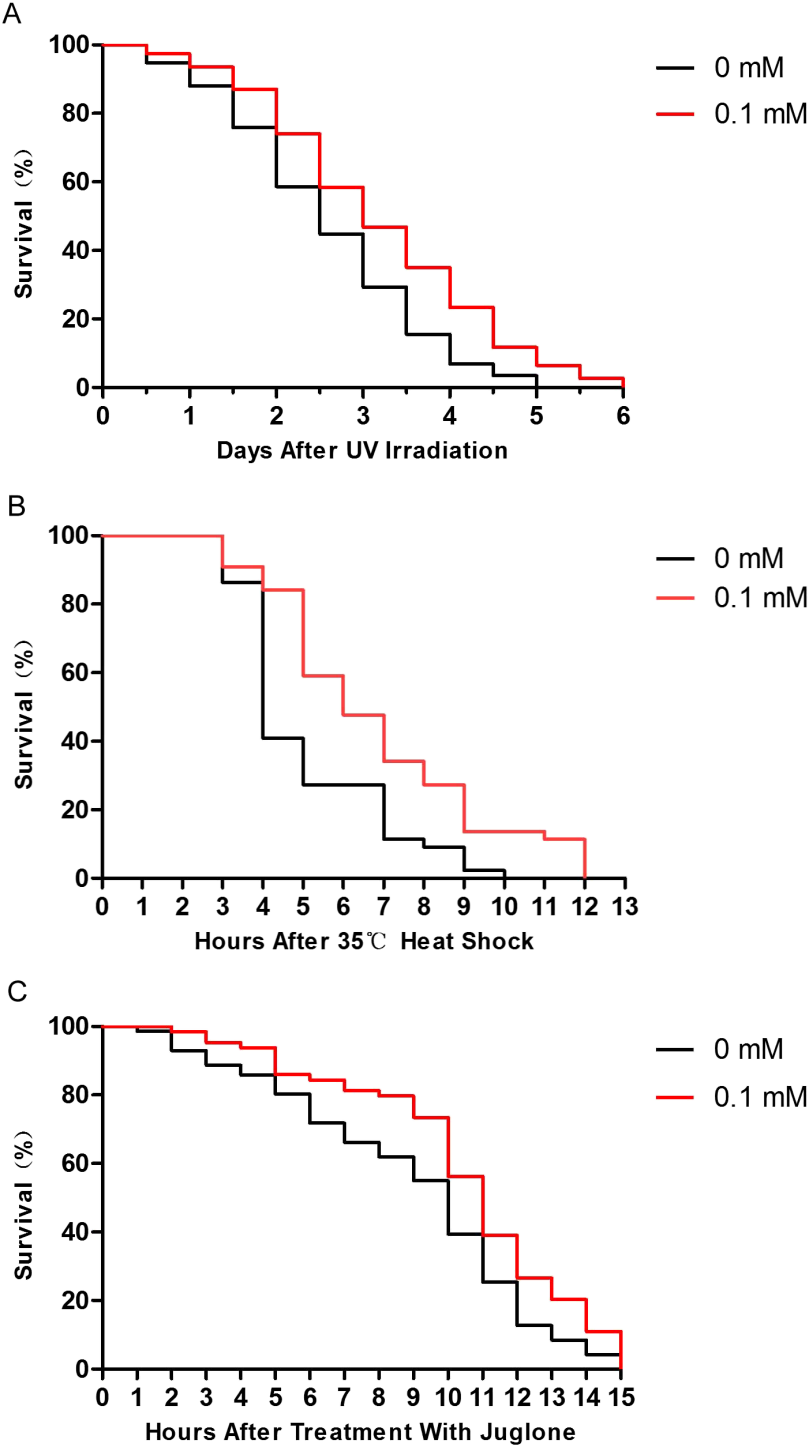
Didymin improved the survival of wild-type *C. elegans* nematodes exposed to various stressors. The adult nematodes were treated with 0.1 mM didymin or DMSO (vehicle control) for 48 h, followed by three types of stressors. Compared to control nematodes, didymin significantly raised the resistance of nematodes to (A) UV irradiation (*n* > 100, *p* < 0.01), (B) heat shock at 35 °C (*n* > 100, *p* < 0.001), and (C) juglone-induced oxidation (*n* > 100, *p* < 0.001).

Many studies have shown that enhancing resistance to stresses can extend the lifespan of *C. elegans* (Chen et al., 2016; Ogawa et al., 2016). Therefore, we performed several stress resistance assays where wild-type nematodes were exposed to various stressors (i.e., 1,000 J/m^2^ of UV irradiation, heat shock at 35 °C, and 200 μM juglone), and the lifespan of nematodes was studied. Compared to control nematodes, the resistance to UV irradiation, thermal, and oxidative stresses increased after treatment with 0.1 mM didymin. Based on our results, the effect of 0.1 mM didymin on UV irradiation was the most obvious in these stress resistance. Thus, we used UV irradiation as the stressor for further studies (Fig. 2).

### Didymin reduces the ROS levels

**Figure 3:**
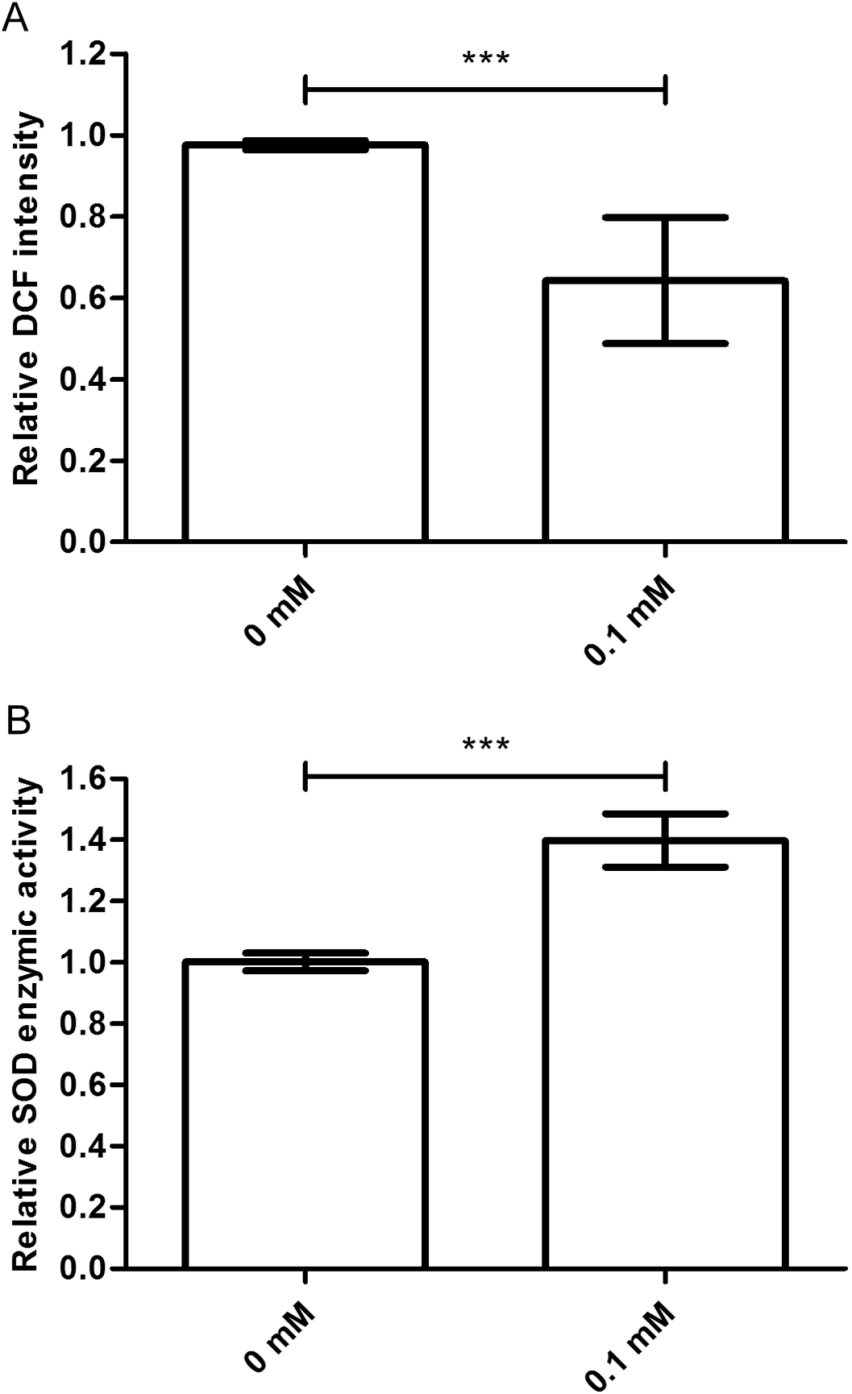
Didymin decreased the ROS levels and increased SOD activity in *C. elegans* nematodes exposed to UV irradiation. The adult nematodes were treated with 0.1 mM didymin for 48 h, followed by UV irradiation. The worms were collected in M9 buffer and tested. Compared to control nematodes, (A) the ROS levels decreased and (B) SOD activity increased in nematodes treated with 0.1 mM didymin (*n* > 10 parallel experiments, *p* < 0.001). The error bars represent SEM (***p < 0.001; 0.001 ≤ **p < 0.01; 0.01 ≤ *p < 0.05).

The ROS levels are associated with the age-related phenotype (Schaar et al., 2015). ROS levels increase when the body is damaged. So, we tested the ROS level in nematodes treated with or without didymin, followed by UV irradiation. Compared to control nematodes, there was a significant decrease in ROS accumulation after UV irradiation in nematodes treated with 0.1 mM didymin. Thus, the resistance effect of didymin on UV irradiation was related to lower ROS (Fig. 3A).

### Didymin increases the SOD activity

Superoxide dismutase is one of the important biological antioxidant enzymes. It eliminates harmful substances during metabolic produces such as ROS (Mi et al., 2018). Compared to control nematodes, we found that SOD activity was higher in nematodes treated with 0.1 mM didymin and exposed to UV irradiation. In other words, SOD activity of didymin might be an important factor in UV irradiation of nematodes (Fig. 3B).

### Didymin acts through the insulin/IGF-1-like signaling pathway in *C. elegans*

**Figure 4:**
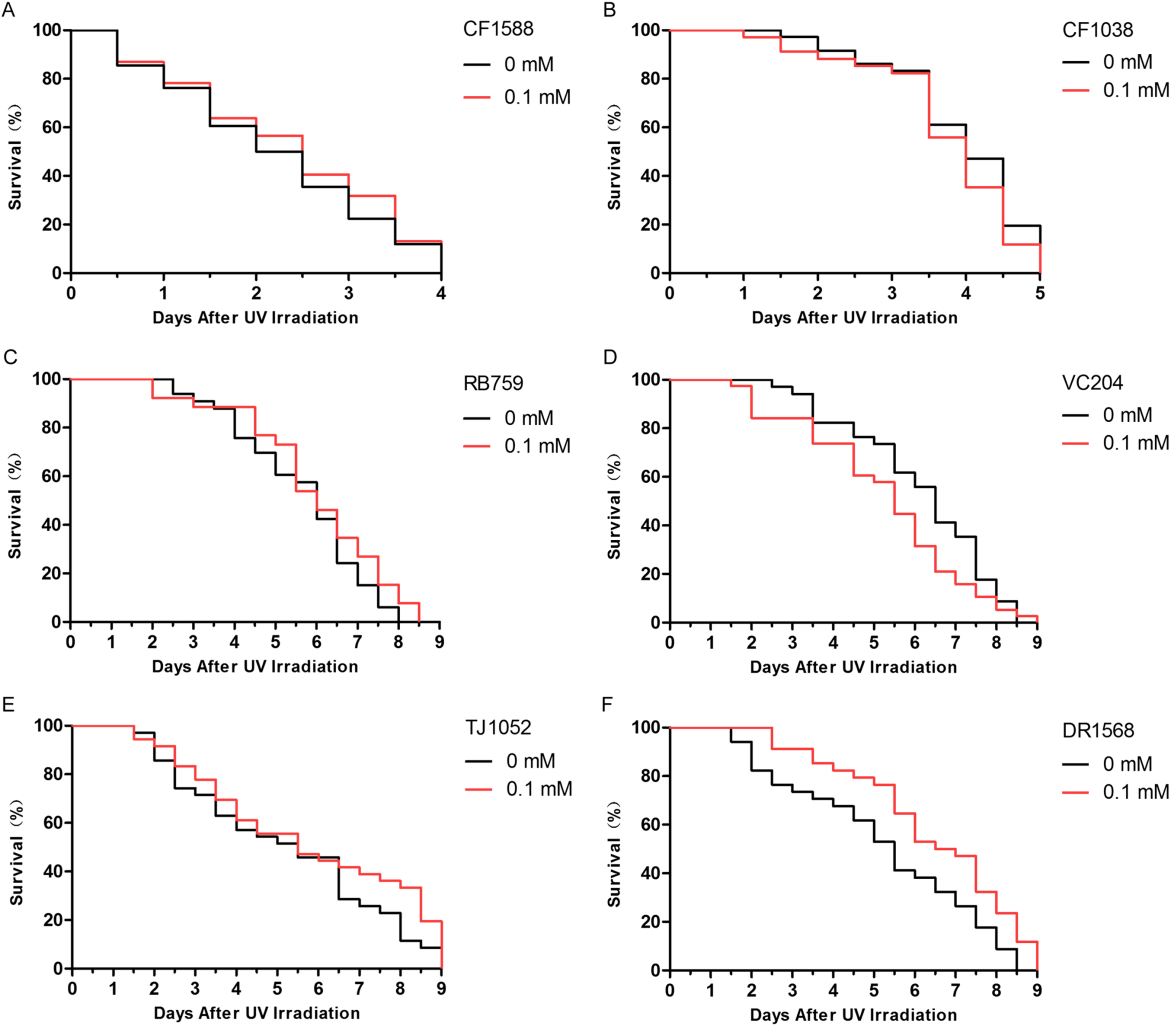
Didymin acted through the insulin/IGF-1-like signaling pathway in *C. elegans*. Six kinds of adult mutant nematodes were treated with 0.1 mM didymin for 48 h, followed by UV irradiation. The didymin-treated mutants (A) *daf-2* and *daf-16* (*n* > 100, *p* > 0.05), (B) *daf-16* (mu86) (*n* > 100, *p* > 0.05), (C) *akt-1* (ok525) (*n* > 100, *p* > 0.05), (D) *akt-2* (ok393) (*n* > 100, *p* > 0.05), and (E) *age-1* (hx546) (*n* > 100, *p* > 0.05) reversed their resistance to UV irradiation. However, the didymin-treated mutant (F) *daf-2* (e1371) (*n* > 100, *p* < 0.001) failed to reverse its resistance to UV irradiation, indicating that didymin might act on DAF-2 to increase the lifespan of *C. elegans* nematodes exposed to UV irradiation.

The insulin/IGF-1-like signaling pathway in nematodes includes key components such as DAF-2, AGE-1, AKT-1, AKT-2, and DAF-16 (Evans, Chen & Tan, 2008). Because *daf-2* and *daf-16* are two important genes that are located upstream and downstream, respectively, in the insulin/IGF-1-like signaling pathway, we judged that didymin-induced lifespan extension after UV irradiation might involve this pathway. We found that didymin certainly failed to enhance resistance to UV irradiation of the *daf-2* and *daf-16* double genes mutant. Moreover, after UV irradiation, there was no obvious difference in *daf-16*, *akt-1*, *akt-2*, and *age-1* single gene mutant exposed to 0.1 mM didymin compared to control nematodes. However, 0.1 mM didymin improved UV irradiation resistance of *daf-2* mutant. Taken together, results showed that didymin improves resistance of nematodes after UV irradiation through the insulin/IGF-like signaling pathway, and it might act on *daf-2* (Fig. 4).

**Figure 5:**
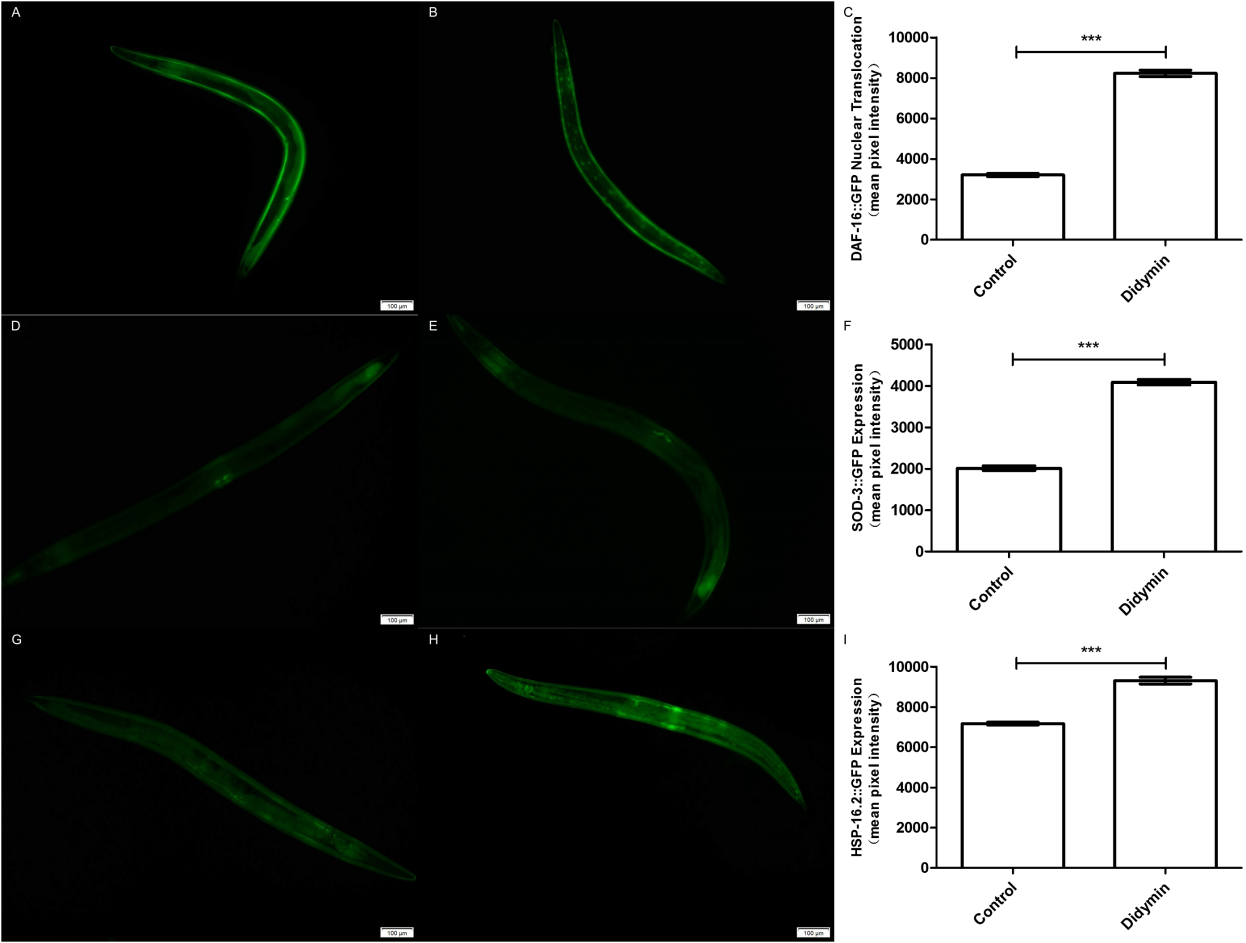
Didymin enhanced the nuclear localization of DAF-16 and the expression of SOD-3 and HSP-16.2 after exposure to UV irradiation. Three GFP transgenic nematodes were exposed to 0.1 mM didymin for 48 h, followed by UV irradiation. Didymin enhanced (A–C) the nuclear localization of DAF-16 (*n* = 10 each group, *p* < 0.001) and the expression of SOD-3 (D–F) (*n* = 10 each group, *p* < 0.001) and HSP-16.2 (G–I) (*n* = 10 each group, *p* < 0.001) after UV irradiation. (A) Control TJ356 worms; (B) TJ356 worms treated with didymin; (D) control CF1553 worms; (E) CF1553 worms treated with didymin; (G) control CL2070 worms; (H) CL2070 worms treated with didymin. The error bars represent SEM (***p < 0.001; 0.001 ≤ **p < 0.01; 0.01 ≤ *p < 0.05).

An additional study was conducted to determine whether the improvement in anti-UV irradiation stress of *C. elegans* pretreated with didymin depended on the regulatory activity of *daf-16*. Didymin’s function on DAF-16 translocation after UV irradiation in *C. elegans* was determined by TJ356 strain, and the expression levels of SOD-3 and HSP-16.2 were detected by CF1553 and CL2070 strains (Meng & Li, 2017; Mukhopadhyay, Oh & Tissenbaum, 2006). As we expected, DAF-16::GFP translocated to the nucleus, and the expression of GFP-fused *sod-3* or *hsp-16.2* was significantly increased after treatment with 0.1 mM didymin (Fig. 5).

**Figure 6:**
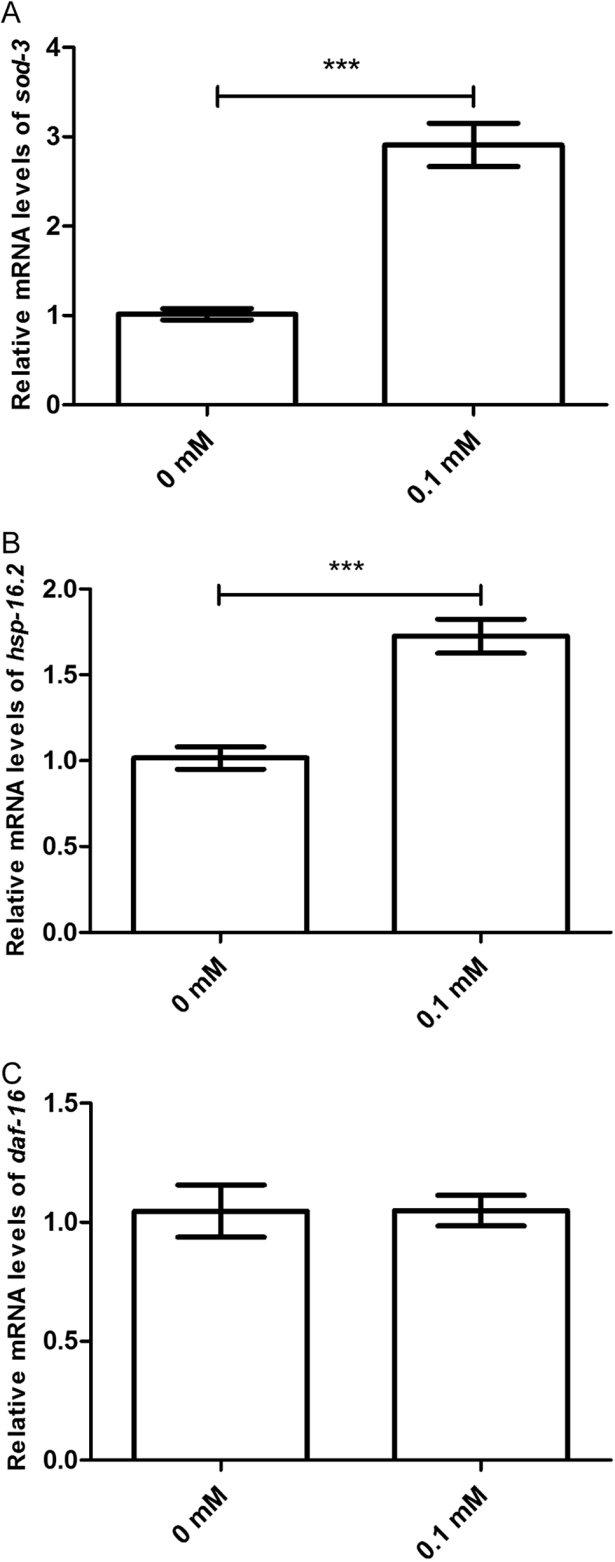
Didymin increased the mRNA expression levels of *sod-3* and *hsp-16.2*, but not that of *daf-16* in nematodes exposed to UV irradiation. Adult wild-type nematodes were treated with 0.1 mM didymin for 48 h, followed by UV irradiation. Didymin significantly increased the mRNA levels of (A) *sod-3* (*p* < 0.001) and (B) *hsp-16.2* (*p* < 0.001). However, there was no change in the mRNA level of (C) *daf-16* after treatment (*p* > 0.05). This experiment was performed at least three times, with 500 nematodes per group. The error bars represent SEM (***p < 0.001; 0.001 ≤ **p < 0.01; 0.01 ≤ *p < 0.05).

Moreover, the effect of didymin after UV irradiation on *daf-2*/*daf-16*-related target genes was examined by qPCR method. The mRNA levels of *sod-3* and *hsp16.2* were significantly increased. However, there was no change in the mRNA level of *daf-16* compared with the control. In summary, data suggest that didymin improves UV irradiation resistance of *C. elegans* through the insulin/IGF-1-like signaling pathway, at least partially (Fig. 6).

## Discussion

Several animal models of longevity have already been established, including *C. elegans*, which have a relatively short lifespan, as well as clear genetic and environmental factors (Kaletta & Hengartner, 2006). With regard to the effects of radiation on nematodes, detailed studies have been conducted at the molecular, cellular, and individual levels for nearly three decades (Sakashita et al., 2010), radiation can damage DNA and produce ROS. Insulin/IGF-1-like signal pathways, which control the lifespan of the nematode, have been discovered and studied in detail. It is found that radiation and oxidative stress can act on the insulin/IGF-1-like signaling pathway, which might lead to accelerated senescence of nematodes and shortened lifespan (Zhou et al., 2018a). Because the signaling system which is related to aging and oxidative stress in nematode cells has been precisely studied, nematodes will provide more help in understanding the mechanisms of higher organisms.

Didymin is a commercially available dietary flavonoid glycoside that is derived from citrus fruits (Singhal et al., 2017). This study confirmed the stress resistance activity of didymin in the nematode model system. First, we observed that didymin could significantly prolong the lifespan of wild-type nematodes in a dose-dependent manner. The most effective concentration of didymin was 0.1 mM dissolved in 0.2% DMSO. Thus, we used 0.1 mM didymin for the remaining experiments in this study.

Lifespan and stress resistance are tightly interrelated (Kenyon, 2010; Larsen, 1993). Thus, we next tested the lifespan of nematodes treated with didymin under different stressors. Stress resistance was measured in terms of lifespan after pressure acceptance. We found that the lifespan of nematodes exposed to various stressors was also extended in animals treated with didymin. These results indicated that didymin played a role in stress resistance.

Both environmental damage and normal cellular metabolism can produce ROS, resulting in cell structure breaking or critical cellular functions losing (Fatouros et al., 2012). SOD activity is a pivotal oxidative stress response that serves to protect living cells from ROS (Oh et al., 2006; Sampayo, Olsen & Lithgow, 2003). Interestingly, we found that nematodes treated with didymin showed low ROS levels and high SOD activity after UV irradiation. Therefore, didymin might resist UV irradiation by reducing ROS levels and increasing SOD activity.

Insulin/IGF-1-like signaling is considered to be the first pathway to regulate lifespan of *C. elegans* (Meng & Li, 2018). In addition, it plays important roles in stress resistance, metabolism, and reproduction (Lee et al., 2003). Insulin-like ligands interact with DAF-2 to activate AGE-1, thereby preventing DAF-16 activation through AKT (AKT-1/2) stimulation (Kenyon, 2011). In this study, mutant nematodes CF1588, CF1038, RB759, VC204, TJ1052, and DR1568 were tested. Our results showed that didymin didn’t change the lifespan of *daf-16*(mu86) & *daf-2*(e1370), *daf-16*(mu86), *akt-1*(ok525), *akt-2*(ok393), and *age-1*(hx546) after UV irradiation compared with control, but it increased resistance in the *daf-2* (e1371), suggesting that didymin might protect nematodes from UV irradiation by regulating the insulin/IGF-1-like signaling pathway, and it might act on *daf-2*.

In order to find the relationship between UV irradiation and protein expression, we employed GFP transgene mutants TJ356, CF1553, and CL2070. DAF-16 nuclear translocation can regulate transcription of many genes which are related with stress tolerance and longevity (Su & Wink, 2015). We found that didymin significantly make more DAF-16 moved to nuclear. *sod-3* works downstream of *daf-16* and it is implicated in detoxification. At the moment, *hsp-16.2* is also located downstream of *daf-16*, and it can hold back the aggregation of unfolded and misfolded proteins. Both *sod-3* and *hsp-16.2* are essential in stress resistance of worms (Murphy et al., 2003). As our expected, data suggested that both SOD-3 and HSP-16.2 were expressed more after didymin treatment and UV irradiation.

To analyze age-related changes in gene expression, we used qPCR. Our results showed that didymin increased mRNA levels of *sod-3* and *hsp-16.2*. However, the mRNA level *daf-16* was unchanged compared with the control. Taken together, these results demonstrated that the effect of didymin on nematodes with regard to UV irradiation stress resistance was at least part through the insulin/IGF-1-like signaling pathway.

## Conclusion

In summary, we found that didymin improved several stress resistance of nematodes. The effect of didymin on UV irradiation resistance might act via the insulin-like signaling pathway. We would like to further study the mechanism of its role in UV irradiation resistance.

## Supplemental Information

10.7717/peerj.6218/supp-1 Supplemental Information 1 Raw data of Figure 1.

10.7717/peerj.6218/supp-2 Supplemental Information 2 Raw data of Figure 2.

10.7717/peerj.6218/supp-3 Supplemental Information 3 Raw data of Figure 3.

10.7717/peerj.6218/supp-4 Supplemental Information 4 Raw data of Figure 4.

10.7717/peerj.6218/supp-5 Supplemental Information 5 Raw data of Figure 5.

10.7717/peerj.6218/supp-6 Supplemental Information 6 Raw data of Figure 6.

## Additional Information and Declarations

### Data Availability

The following information was supplied regarding data availability: The raw measurements are provided in the Supplementary Files.
